# SUCCESSFUL AGING SCALE DEVELOPMENT FOR COMMUNITY-DWELLING OLD-OLD (75+) IN CHINA: A PRELIMINARY ANALYSIS

**DOI:** 10.1093/geroni/igz038.1331

**Published:** 2019-11-08

**Authors:** Minzhi Ye, Lin Chen, Lin Gu

**Affiliations:** 1 Benjamin Rose Institute on Aging, Cleveland, Ohio, United States; 2 Department of Social Work, Fudan University, Shanghai, Shanghai, China (People's Republic); 3 Shanghai Volunteer Guidance Center for Longevity, Shanghai, Shanghai, China (People's Republic)

## Abstract

Thousands of articles have discussed the concept of successful aging, yet no existed scale to measure its components among old-old (age 75+) in urban China. This study used a mixed method to develop a valid scale in Shanghai from July 2016 to Nov 2018. We first conducted 97 semi-structured, in-depth interviews on community-dwelling old-old and listed 70 items based on the findings and previous studies (e.g., Lubben, 1988). Ten multidisciplinary professionals and eight old-old had focus groups to evaluate the content validity of the scale. We used the final 22 items to survey 208 community-dwelling old-old in twelve neighborhoods. The mean age is 81, with 40% females. The principal component analysis showed a 13-item scale representing four domains (self-reliant, aging attitude, self-value expression, and social support), with 0.67 total reliability (Cronbach's Alpha). This study provided a set of quality indicators to evaluate old-old’s life in urban China.

